# Supplementary material for: Coordinating cell polarization and morphogenesis through mechanical feedback
Source: PLoS Comput Biol. 2021 Jan 28;17(1):e1007971. doi: 10.1371/journal.pcbi.1007971 (PMC7872284; doi:10.1371/journal.pcbi.1007971)
Supplement: S1 File — Parameters values for the minimal coarse-grained description and details on the 3D stochastic simulations. (PDF) [file pcbi.1007971.s001.pdf]

Supporting Information

Coordinating cell polarization and morphogenesis through mechanical feedback

Samhita P. Banavar, Michael Trogdon, Brian Drawert,

Tau-Mu Yi,Linda R. Petzold & Otger Campàs

Physical parameters for course grained model. These values are part of the dimensionless parameters of the course grained model

**Parameter**	**Description**	**Value**
P	Turgor pressure of budding yeast	0.6 ± 0.2 MPa [1]
ρ_w	Density of 1,3-β glucans in cell wall	–
μ_0	Apical viscosity of cell wall	–
k_s	Rate of new wall synthesis	–
ρ_0	Membrane concentration of Bni1	–
k_{D}	Apical rate of endocytosis	$0.02 \pm 0.02 s^{-1}$ [2]
		$0.027 s^{-1}$ [3]
k_{X}	Apical rate of exocytosis	$0.045 s^{-1}$ [3]
k_{R}	Recruitment rate of Bni1 by Cdc42	$1.6\ 10^{-6} s^{-1}$ [4]
k_{I}	Inactivation rate of Bni1	$0.018 s^{-1}$ [4]
D	Membrane diffusion constant of Cdc42	$0.0053 \mu m^2 s^{-1}$ [4]

Spatial stochastic polarization model details and parameter values Reactions for the spatial stochastic model of Cdc42 and actin polarization coupled to cell wall mechanics presented in the main text. Species with a subscript 'm' (e.g. $Cdc42GDP_m$) refer to membrane bound species and species with a subscript 'c' (e.g. $Cdc42GDP_c$) refer to cytoplasmic species.

$$Cdc42GDP_c \xrightarrow{\beta_2} Cdc42GDP_m$$
$$Cdc42GDP_m \xrightarrow{\beta_3} Cdc42GDP_c$$
$$Cdc42GDP_c + Actin_m \xrightarrow{\beta_1} Cdc42GDP_m + Actin_m$$
$$Cdc42GDP_m + Actin_m \xrightarrow{\alpha_1} Cdc42GTP_m + Actin_m$$
$$Cdc42GTP_m \xrightarrow{\alpha_2} Cdc42GDP_m$$
$$Actin_c + Cdc42GTP_m \xrightarrow{A_{on}} Actin_m + Cdc42GTP_m$$
$$Actin_c \xrightarrow{A_{\mathrm{CWI}}[\dot{\epsilon}_s + \dot{\epsilon}_\phi]} Actin_m$$
$$Actin_m \xrightarrow{A_{off}} Actin_c$$

Parameter	Value	Description	Source
D_m	0.0053 $\mu m^2 s^{-1}$	Diffusion constant on membrane	[4]
D_a	0.0 $\mu m^2 s^{-1}$	No actin diffusion on membrane	[4]
D_c	10 $\mu m^2 s^{-1}$	Diffusion constant in cytoplasm	[5]
R	2 μm	Radius of cell	[4]
N_C	3000	Total number of Cdc42 molecules	[6]
N_A	40	Total number of Actin cables	[4]
α_1	0.2 $\mu m^2 s^{-1}$	Activation of Cdc42 by Cdc24	[6]
α_2	1 s^{-1}	Deactivation of Cdc42	[6]
β_1	0.266 $\mu m^3 s^{-1}$	Activation of Cdc42 by Cdc24	[6]
β_2	0.28 $\mu m s^{-1}$	Attachment of Cdc42 to membrane	[6]
β_3	1 s^{-1}	Detachment of Cdc42 from membrane	[6]
A_{on}	0.197 $\mu m^3 s^{-1}$	Recruitment of Actin by Bni1	[4]
A_{off}	2.70 s^{-1}	Detachment of Actin from membrane	[4]
A_{CWI}	varied	Cooperativity of mechanical feedback	
$\dot{\epsilon}_s + \dot{\epsilon}_\phi$		Cell wall strain rates	

References

[1] Schaber J, Angel Adrover M, Eriksson E, Pelet S, Petelenz-Kurdziel E, Klein D et al. Biophysical properties of *Saccharomyces cerevisiae* and their relationship with HOG pathway activation. Eur Biophys J. 2010; 39(11):1547–1556.

[2] Jose M, Tollis S, Nair D, Sibarita J, McCusker D. Robust polarity establishment occurs via an endocytosis-based cortical corralling mechanism. The Journal of Cell Biology. 2013; 200(4): 407–418.

[3] Carrillo L, Cucu B, Bandmann V, Homann U, Hertel B, Hillmer S, et al. High-Resolution Membrane Capacitance Measurements for Studying Endocytosis and Exocytosis in Yeast. Traffic. 2015; 16(7): 760–772.

[4] Lawson MJ, Drawert B, Khammash M, Petzold L, Yi TM. Spatial Stochastic Dynamics Enable Robust Cell Polarization. PLOS Computational Biology. 2013;9(7):1–12. doi:10.1371/journal.pcbi.1003139.

[5] Slaughter BD, Schwartz JW, Li R. Mapping dynamic protein interactions in MAP kinase signaling using live-cell fluorescence fluctuation spectroscopy and imaging. Proceedings of the National Academy of Sciences. 2007;104(51):20320–20325. doi:10.1073/pnas.0710336105.

[6] Klünder B, Freisinger T, Wedlich-Söldner R, Frey E. GDI-Mediated Cell Polarization in Yeast Provides Precise Spatial and Temporal Control of Cdc42 Signaling. PLOS Computational Biology. 2013;9(12):1–12. doi:10.1371/journal.pcbi.1003396.
